# Extremely thick cell walls and low mesophyll conductance: welcome to the world of ancient living!

**DOI:** 10.1093/jxb/erx045

**Published:** 2017-04-13

**Authors:** Linda-Liisa Veromann-Jürgenson, Tiina Tosens, Lauri Laanisto, Ülo Niinemets

**Affiliations:** 1 Institute of Agricultural and Environmental Sciences, Estonian University of Life Sciences, Kreutzwaldi 1, Tartu 51014, Estonia; 2 Estonian Academy of Sciences, Kohtu 6, 10130 Tallinn, Estonia

**Keywords:** Conifer, *g*_m_, gymnosperm, LES, LMA, nitrogen, photosynthesis.

## Abstract

Mesophyll conductance is thought to be an important photosynthetic limitation in gymnosperms, but they currently constitute the most understudied plant group in regard to the extent to which photosynthesis and intrinsic water use efficiency are limited by mesophyll conductance. A comprehensive analysis of leaf gas exchange, photosynthetic limitations, mesophyll conductance (calculated by three methods previously used for across-species comparisons), and the underlying ultra-anatomical, morphological and chemical traits in 11 gymnosperm species varying in evolutionary history was performed to gain insight into the evolution of structural and physiological controls on photosynthesis at the lower return end of the leaf economics spectrum. Two primitive herbaceous species were included in order to provide greater evolutionary context. Low mesophyll conductance was the main limiting factor of photosynthesis in the majority of species. The strongest sources of limitation were extremely thick mesophyll cell walls, high chloroplast thickness and variation in chloroplast shape and size, and the low exposed surface area of chloroplasts per unit leaf area. In gymnosperms, the negative relationship between net assimilation per mass and leaf mass per area reflected an increased mesophyll cell wall thickness, whereas the easy-to-measure integrative trait of leaf mass per area failed to predict the underlying ultrastructural traits limiting mesophyll conductance.

## Highlight

Low mesophyll conductance was the most important limiter of photosynthesis in the majority of evolutionarily old species and resulted from thick cell walls and low exposed chloroplast area.

## Introduction

The 36-fold variation of net photosynthetic rate across C3 species (Wright *et al.*, 2004) cannot be explained only by stomatal restrictions and biochemical potentials, because the CO_2_ diffusion efficiency from substomatal cavities to chloroplasts (mesophyll diffusion conductance; *g*_m_) plays an important role in shaping photosynthetic capacities and leaf resource-use efficiency across Earth’s ecosystems (Warren *et al.*, 2003; Niinemets *et al.*, 2009*b*, 2011; Terashima *et al.*, 2011; Tosens *et al.*, 2012*b*, 2016). The general consensus is that ignoring *g*_m_ in carbon gain calculations prevents the correct simulation of photosynthesis due to biased maximum velocity of carboxylation especially in stressful field environments (Manter and Kerrigan, 2004; Niinemets *et al.*, 2011). Still, *g*_m_ has been ignored in carbon gain calculations because of our lack of knowledge about its various causes and the main drivers of its variation across species (Niinemets *et al.*, 2009*a*).

Mesophyll conductance has now been estimated for more than 100 species from all major plant groups. Available data show that considerable variation of *g*_m_ between plant groups exists and it is larger than that of stomatal conductance (*g*_s_) (Flexas *et al.*, 2012; Tosens *et al.*, 2016). *g*_m_ depends on the length and characteristics of the CO_2_ diffusion path from substomatal cavities to chloroplasts (Evans *et al.*, 2009; Hassiotou *et al.*, 2009; Terashima *et al.*, 2011; Tosens *et al.*, 2012*b*). The main limitations to CO_2_ diffusion are in the mesophyll liquid phase. Two traits, mesophyll cell wall thickness (*T*_cwm_) and chloroplast surface area exposed to intercellular airspace (*S*_c_/*S*), have been highlighted as the strongest limiting factors of *g*_m_, but these anatomical traits are highly variable among species (Evans *et al.*, 2009; Terashima *et al.*, 2011; Tosens *et al.*, 2012*b*). In addition, recent studies suggest that chloroplast thickness is also an important barrier limiting diffusion of CO_2_ to Rubisco (Tosens *et al.*, 2012*a*,*b*, 2016; Peguero-Pina *et al.*, 2012; Tomás *et al.*, 2013). The reduction of photosynthesis due to *g*_m_ can be about 25% for mesophytic species and up to 75% in evergreen species (Niinemets *et al.*, 2011). The latter have more robust leaves, e.g. thicker cell walls due to their adaptations to their specific environmental stressors (Niinemets, 2016). Contrarily, ferns and allies have seemingly soft leaves and low leaf mass per area (LMA), but the extent of the photosynthetic limitation by *g*_m_ and net assimilation are in a similar range to that reported for sclerophyllous angiosperms due to their very thick mesophyll cell walls and very low *S*_c_/*S* ([Bibr CIT0044][Bibr CIT0048],*c*; Tosens *et al.*, 2016). That is, *g*_m_ and its underlying traits cannot be simply extended across plant groups, and additional factors such as evolutionary age can play an important role in its determination.

Across spermatophytes *g*_m_ is lowest in gymnosperms (Flexas *et al.*, 2012). Six-fold variation in *g*_m_ has been shown among the 13 conifer species studied so far (Warren *et al.*, 2003; De Lucia *et al.*, 2003; Manter and Kerrigan, 2004; Black *et al.*, 2005; Mullin *et al.*, 2009; Peguero-Pina *et al.*, 2012, 2016). Collectively, the available data suggest that *g*_m_ is an important limiting factor of photosynthesis across and within conifer species. Despite its undoubted importance, only Peguero-Pina *et al.* (2012, 2016) have investigated *g*_m_ together with the underlying structural correlates. Therefore, integrative approaches are needed to characterize the nature of *g*_m_ and its importance in controlling realized photosynthesis rates in gymnosperms.

The leaf economics spectrum (LES) is a dimension of ecological variation reflecting differences across species in the cost of the investment in a unit of leaf area and the return on that investment (Wright *et al.*, 2004). Despite the broad use of LES relationships in generalizing global leaf structure–function relationships, the morpho-physiological basis underlying given trade-offs are poorly understood, especially for species placed at the low-return end (Shipley *et al.*, 2006; Niinemets *et al.*, 2009*a*,b). These species are characterized by high LMA and robust structure, and therefore the realized photosynthetic rates are modified in an important way by leaf morphology (Flexas *et al.*, 2008; Hassiotou *et al.*, 2009; Niinemets *et al.*, 2011; Tosens *et al.*, 2012*b*). For example, high LMA could be associated with high net assimilation rate (*A*_area_) if its main driver is great thickness of mesophyll and high *S*_c_/*S*, or with low *A*_area_ if it is connected with its underlying anatomical variations such as thick mesophyll cell walls (Hassiotou *et al.*, 2009; Tomás *et al.*, 2013). However, the integrative trait of LMA may fail for species like Australian *Proteaceae*, which have mesophytic mesophyll tissue embedded in highly sclerophytic tissue, or in ferns and allies, which have low LMA but high *T*_cwm_ resulting in low photosynthesis (Niinemets *et al.*, 2009*c*; Tosens *et al.*, 2012*b*, 2016). Understanding the underlying ultrastructural traits controlling the global LMA–photosynthesis–nitrogen relationships is inescapable for understanding photosynthetic patterns across Earth’s ecosystems.

The reasons for lower photosynthesis rates and intrinsic water use efficiency (WUE_i_) in gymnosperms are currently poorly known due to lack of studies separating mesophyll and stomatal diffusional and mesophyll biochemical photosynthetic limitations. Information about *g*_m_ with its underlying structural traits is especially limited. Here we set out to characterize the spectrum of photosynthetic strategies and related physiological and morphological traits in gymnosperms positioned at the lower return end of the leaf economics spectrum based on 11 gymnosperm and two evolutionarily older species, *Psilotum nudum* and *Selaginella uncinata*, in order to enhance the evolutionary context. Overall, the included species are evolutionarily old with a phylogenetic age extending from 306 million years for *Psilotaceae* to 75 million years for the genus *Podocarpus* (Pryer *et al.*, 2004; Biffin *et al.*, 2012). *Metasequoia glyptostroboides*, *Cycas revoluta*, *Macrozamia riedlei* and *Araucaria heterophylla* can even be regarded as living fossils as their morphology and habitat have a high resemblance to fossils dated to the Mesozoic (>110 My ago) and even to the late Paleozoic (300 My ago) (e.g. Norstog and Nicholls, 1997; Rydin *et al.*, 2004; Zhang *et al.*, 2015). The specific aims of the study were (i) to analyse the photosynthetic limitations to understand if diffusional limitations, especially *g*_m_, are the most substantial constraints in evolutionarily old plants; (ii) to assess which structural traits are responsible for low CO_2_ diffusion conductance in evolutionarily old species; and (iii) to understand the morpho-physiological basis underlying LES traits in evolutionarily old species.

## Materials and methods

### Plant material and growth conditions

**Fig. 1.**
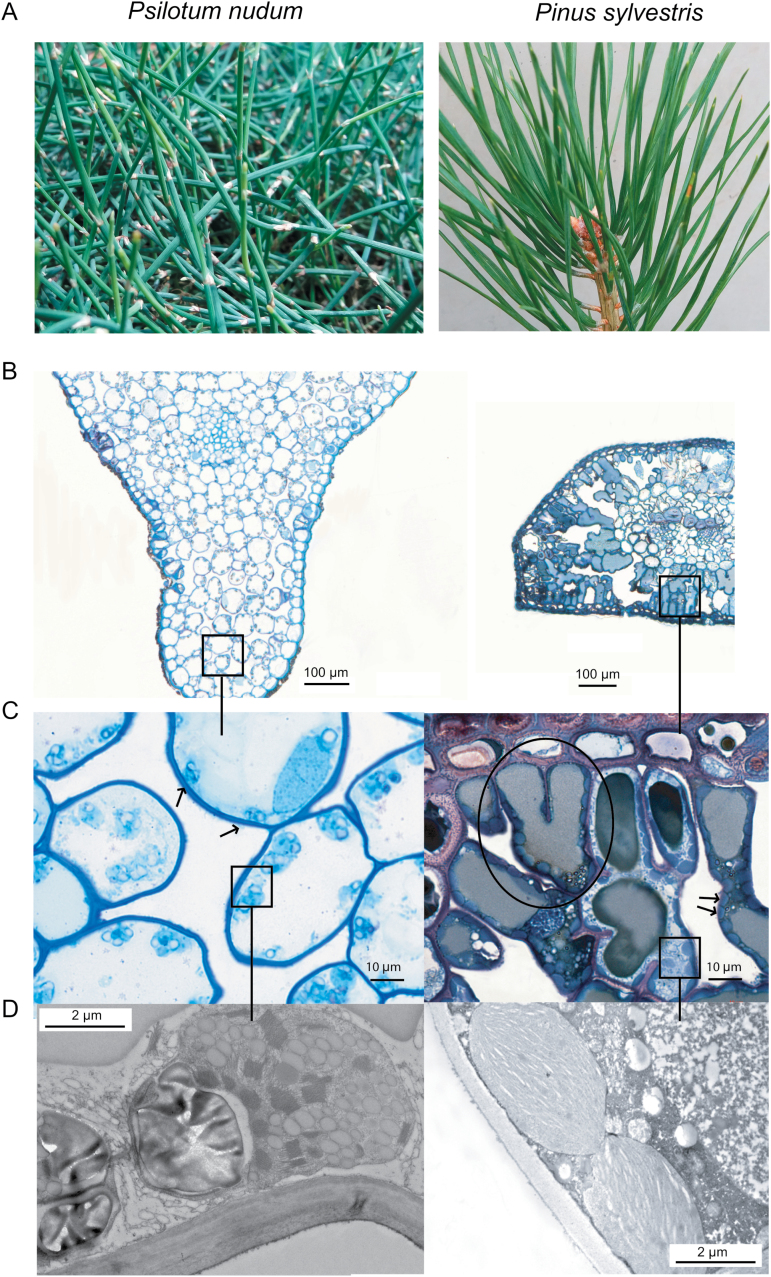
(A, B) Photographs (A) and cross-sections (B) of *Psilotum nudum* (left) and *Pinus sylvestris* (right) to illustrate different leaf internal anatomies. (C,D) Low (*P. nudum*, left) and high (*P. sylvestris*, right) chloroplast area exposed to intercellular airspace (*S*_c_/*S*). Arrows in (C) indicate sparsely (left) and tightly packed chloroplasts (right). The *P. sylvestris* cross-section exhibits lobed cells (circled in C).

Thirteen evolutionarily old species (Fig. 1 and Supplementary Table S1 at *JXB* online) with widely varying shape, size, longevity and structure of photosynthetic organs covering a broad range of evolutionary ages were included in the analysis. Out of the selected species, 11 (*Araucaria heterophylla*, *Cupressus sempervirens*, *Cycas revoluta*, *Ephedra minuta*, *Macrozamia riedlei*, *Metasequoia glyptostroboides*, *Picea abies*, *Pinus sylvestris*, *Podocarpus alpinus*, *Podocarpus nivalis* and *Taxus baccata*) were gymnosperms from three of the four gymnosperm divisions. Two primitive herbaceous plant species (a whisk fern, *Psilotum nudum*, and a clubmoss, *Selaginella uncinata*) were also included.

Five-month-old saplings of *M. glyptostroboides*, *P. alpinus*, *P. nivalis*, *S. uncinata* and *T. baccata* were obtained in October 2014 from a local nursery and transferred to a controlled-conditions phytotron, where they grew for 2–4 months before the measurements were begun. Other species were grown in the phytotron from seeds or seedlings brought from their country of origin (see Supplementary Table S1). All measured leaves emerged and matured in the phytotron conditions. Relative humidity was maintained at 60% and air temperature at 25 °C/22 °C (day/night). Light was provided by high pressure sodium lamps (400 W, E40 tubular clear, Beijing Luxram Lighting Ltd, China) for a 13 h photoperiod. The photosynthetic photon flux density (*Q*) incident to the plants was maintained at 1000 µmol m^–2^ s^–1^ reflecting the natural growth light conditions of the species (Supplementary Table S1). Plants were watered every second day with tap water and fertilized with soluble fertilizer once a week (Substral Miracle for evergreens, N:P_2_O_5_:K_2_O:MgO 23:9:12:2 with microelements). In all cases, fully mature non-senescent foliage was used for the measurements.

### Foliage gas exchange measurements and estimation of mesophyll conductance

Simultaneous gas exchange and fluorescence measurements were conducted with a GFS-3000 portable photosynthesis system (Walz GmbH, Germany). Foliage elements were arranged side by side in the leaf chamber to avoid any overlap and a digital photograph was taken for estimation of the exact projected foliage area enclosed in the chamber after its closure. In all measurements, vapor pressure deficit was kept at *ca* 1.5 kPa, and leaf temperature, measured with a thermocouple, at 25 ºC. Leaf net photosynthesis, the steady-state net photosynthesis rate under saturating *Q* of 1000 µmol m^–2^ s^–1^ (90% red and 10% blue light) and 400 µmol CO_2_ mol^–1^ (atmospheric CO_2_ concentration, *C*_a_), was recorded after the stomata opened and leaf gas exchange rates reached maximum steady state, 30–60 min after the enclosure of foliage in the chamber. Net assimilation *vs*. ambient CO_2_ response curves were measured at a *Q* of 1000 µmol m^–2^ s^–1^ by varying *C*_a_ between 50 and 2000 µmol CO_2_ mol^–1^. After each steady-state net assimilation rate had been estimated, the steady-state fluorescence level (*F*) was recorded and a saturating pulse was given by the leaf chamber fluorimeter of the GFS-3000 system (PAM-fluorometer 3055-FL) to measure the maximum fluorescence yield (*F*_m_′) and estimate the effective photosystem II quantum yield (Φ_PSII_) as (*F*_m_′–*F*)/*F*_m_′ (Genty *et al.*, 1989). The area of the leaf chamber was 8 cm^2^. Once the measurements for a CO_2_ response curve were completed, the light was switched off and the mitochondrial respiration rate (*R*_n_) was measured after a minimum of 30 min of dark adaptation. *g*_s_ and intercellular CO_2_ concentration (*C*_i_) were calculated according to von Caemmerer and Farquhar (1981).

Mesophyll conductance was calculated according to Harley *et al.* (1992):

$$g_{\text{m}}=\frac{A_{\text{area}}}{C_{\text{i}}-\frac{\Gamma *J_{\text{ETR}}+8(A_{\text{area}}+R_{\text{d}})}{J_{\text{ETR}}-4(A_{\text{area}}+R_{\text{d}})}}$$ (1)

where *A*_area_ is the net assimilation rate, *J*_ETR_ is the rate of photosynthetic electron transport derived from chlorophyll fluorescence measurements, *C*_i_ is the CO_2_ concentration in sub-stomatal cavities, *R*_d_ is the rate of non-photorespiratory respiration in the light and Γ* is the hypothetical CO_2_ compensation point without *R*_d_. *R*_d_ was estimated as half of *R*_n_ (Niinemets *et al.*, 2005). This common adaptation is supported by previous experimental observations (Villar *et al.*, 1995; Piel *et al.*, 2002). Γ* was taken as 42.9 μmol mol^–1^ at 25 °C (Bernacchi *et al.*, 2001) as it has been shown before on vastly variable plant functional groups and *g*_m_ values that there is no significant difference between *g*_m_ calculated with Γ* according to Galmés *et al.* (2005) and Bernacchi *et al.* (2001) (Tomás *et al.*, 2013). *J*_ETR_ was estimated from non-photorespiratory conditions (2% oxygen) from an *A*–*C*_i_ curve, although the calculations of *g*_m_ were relatively insensitive to moderate variations in *J*_ETR_ (<0.5%; see Niinemets *et al.*, 2005). Therefore, the main assumption of Harley’s variable *J* method was not breached. The data were corrected for chamber leaks according to Flexas *et al.* (2007*a*) and Rodeghiero *et al.* (2007) using empty chamber measurements. Leaf absorption was measured with an integrating sphere (leaf absorption coefficient varied between 0.84–0.90). Gaskets were changed regularly, and modelling paste and paraffin film were used with thick leaves to ensure no leakage. Mesophyll conductance was calculated from measurements of net assimilation rate over the *C*_i_ range of 150–350 μmol mol^–1^, because the *g*_m_ values are stable over this range and its estimates are relatively insensitive to minimal Γ*, *R*_d_ and *A* errors (Harley *et al.*, 1992; Niinemets *et al.*, 2006). WUE_i_ was defined as *A*_area_/*g*_s_ (Flexas *et al.*, 2013). As some plants studied here have small leaves or needles growing on several sides of the branch, not all of these were perpendicular to the light source. However, this is also the case for when they are growing in natural conditions as the branches were positioned in the same direction to the light source as they had grown.

Additionally, the one-dimensional within-leaf gas diffusion model of Niinemets and Reichstein (2003) was applied to provide an anatomically based estimate of *g*_m_ as in Tosens *et al.* (2012*b*) and from *A*–*C*_i_ curves as in Ethier and Livingston (2004).

### Quantitative limitation analyses of *A*_area_ and partial limitations of *g*_m_

The relative controls on *A*_area_ imposed by stomatal conductance (*l*_s_), mesophyll conductance (*l*_m_), and biochemical capacity (*l*_b_) were calculated following the Grassi and Magnani (2005) approach, as has been successfully used for a large number of highly variable species from lycophytes, horsetails and ferns to Australian sclerophylls in Tomás *et al.* (2013) and Tosens *et al.* (2012*b*, 2016). These three values sum to 100% and characterize the extent to which any of the three limitations curbs photosynthesis at the given values of the other two. The contribution of different components of cellular resistance to total cellular resistance to CO_2_ diffusion was estimated from the anatomical model following Tosens *et al.* (2016). This share of limitation (*l*_i_) by different liquid phase components was calculated as:

$$l_{\text{i}}=\frac{g_{\text{m}}}{g_{\text{i}}\cdot \frac{S_{\text{c}}}{S}}$$ (2)

where *l*_i_ is the limitation by the cell wall, the plasmalemma, cytosol, chloroplast envelope and stroma, and *g*_i_ refers to the diffusion conductance of each corresponding diffusion pathway. The limitation of each cellular component was scaled up with *S*_c_/*S*.

### Estimation of leaf dry mass per area and nitrogen content

Foliage used for gas exchange measurements was further used for anatomical, morphological and chemical measurements. Individual foliage elements (leaves, needles or scales) were removed from the twigs and scanned at 300 dpi. The foliage was thereafter oven-dried at 70 °C for 48 h, and its dry mass was determined. Leaf projected area was determined from scanned images with ImageJ 1.48v software (Wayne Rasband/NIH, Bethesda, MD, USA) or calculated from the measured length and widths of sides measured with digital precision calipers (Mitutoyo CD-15DC, Mitutoyo Ltd, Andover, UK) for needles and cladodes (Niinemets *et al.*, 2007). From these measurements, LMA and density (*D*_leaf_=LMA/*T*_leaf_) were calculated (Niinemets, 1999; Poorter *et al.*, 2009). Nitrogen content per dry mass (*N*_mass_) was determined by the dry combustion method with a Vario MAX CNS elemental analyser (Elementar, Hanau, Germany), and leaf nitrogen content per area as *N*_mass_/LMA.

### Light and electron microscopy

A sub-sample of leaves used for gas exchange measurements was taken for leaf anatomical measurements. Light and electron microscopy sample preparation and analyses followed Tosens *et al.* (2012*b*). Six samples were taken from three specimens of each species. Foliage cuts of approximately 6 × 4 mm (or width of the needle or cladode according to species) were taken from intercostal areas. In *C. sempervirens* scales and in *S. uncinata* whole leaves were stripped from the branches with tweezers. The plant material was infiltrated in a fixation buffer (3% glutaric aldehyde and 2% paraformaldehyde in 0.1 M phosphate buffer, pH 6.9) under vacuum in a syringe. The samples were post-fixed for 1 h in an osmium tetroxide solution (2%) and dehydrated in a series of increasingly stronger ethanol solutions and embedded in LR white resin (Electron Microscopy Sciences, Hatfield, PA, USA) according to standard procedures (Tosens *et al.*, 2012*b*). Subsequently, the samples were polymerized in an oven at 60 °C for 26 h. Semi-thin cross-sections of 1 µm for light microscopy, and ultra-thin cross-sections of 70 nm were prepared by an ultramicrotome (Leica EM UC7, Leica Vienna, Austria) for transmission electron microscopy (TEM). The semi-thin sections were stained with toluidine blue for light microscopy, and the sections for TEM were stained with lead citrate and then mounted on formvar–carbon covered copper meshes (Electron Microscopy Sciences). Stained semi-thin sections were viewed in bright field ensuring that all sections were ideally straight with a Nikon Eclipse E600 microscope with phase contrast at magnifications of ×100, ×200 and ×400 and photographed with a Nikon 5 MP digital microscope camera DS-Fi1 (Nikon Corp., Kyoto, Japan). A Philips Tecnai 10 TEM (FEI, Eindhoven, Netherlands) was used to view the ultra-thin sections with the accelerating voltage of 80 kV and magnification between ×1800 and ×14 000. Between five and seven fields of view per sample were measured.

From digital images the following parameters were measured: fraction of intercellular airspace (*F*_ias_), leaf thickness from adaxial to abaxial cuticle (*T*_leaf_), mesophyll thickness excluding vascular bundles (*T*_mes_), cytoplasm thickness (*T*_cyt_), chloroplast thickness (*T*_chl_), mesophyll cell wall thickness (*T*_cwm_), chloroplast and mesophyll cell wall area from which *S*_c_/*S* and mesophyll area exposed to intercellular airspace (*S*_m_/*S*) were calculated according to Evans *et al.* (1994) (Table 2). These characteristics were measured for at least ten spongy and ten palisade parenchyma cells and tissue-volume weighted averages were calculated in the species with distinctive separation of mesophyll to spongy and palisade parenchyma. Thirty cells were analysed for every specimen. Light and electron micrographs were analysed with ImageJ 1.48v software.

### Statistical analyses

For each species, all measurements were replicated at least with three individual plants. Linear and non-linear regression analyses, *t*-tests and ANCOVA were used to examine the relationships among the traits and test for trait differences among species using Statistica 10.0 (StatSoft Inc., Tulsa, OK, USA). The choice between linear and non-linear models depended on the shape of the relationships, degree of explained variance and normality of data and residuals, and models providing the greatest *r*^2^ and lowest deviations from normality of residuals were used. Due to high variability of foliage shapes and structures, individual species were occasionally outliers in statistical relationships, especially in relationships exploring the effects of individual underlying traits on composite variables. For instance, some correlations were significant when the gymnosperms were considered, but not when the spore-bearing species were included. We denote these and analogous outliers separately in bivariate statistical relationships.

### Phylogenetic analyses

Sequences of the plant barcode genes maturase K (*matK*; a gene that encodes an intron splicing protein) and *RbcL* (a gene that encodes the large subunit of Rubisco; both genes are in the chloroplastic DNA; Hollingsworth *et al.*, 2009) were extracted from NCBI GenBank (www.ncbi.nlm.nih.gov, last accessed July 2015). This information was not available for *Macrozamia riedlei*, so the closest available relative, *M. moorei*, was used. Phylogenetic analyses were conducted and phylogenetic trees were created by the neighbor-joining method using standard procedures in MEGA6 (Tamura *et al.*, 2013). The maximum composite likelihood model was used for estimates of evolutionary divergence (Tamura *et al.*, 2004), and the maximum likelihood method based on the Tamura–Nei model (Tamura and Nei, 1993) was used to create phylogenetic topology with the superior log-likelihood value. The evolutionary age of genera was based on previously published literature (Table 1: smaller numbers indicate evolutionarily closer species). This information was correlated with the study parameters using *t*-tests and ANCOVA. The Akaike information criterion was used as a measure of the relative quality of the multiple linear models used here.

**Table 1.** *Phylogenetic ages of the genera*, *growth form*, *leaf habit and distances of studied taxa and estimates of evolutionary divergence* Species are in the order of their evolutionary age. Evolutionary divergence is defined as the number of base substitutions per site between sequences.

Species (abbreviation)	Family	Evolutionary age of genus (My)	Growth form	Evolutionary divergence											
*Selaginella uncinata* (*S.u.*)	Selaginellaceae	405 (Lang *et al.*, 2010)	Herb	*S.u.*	*Ps.n.*	*A.h.*	*C.r.*	*Ma.r.*	*E.m.*	*Me.g.*	*Pin.s.*	*Pic.a.*	*T.b.*	*Po.a.*	*Po.n.*
*Psilotum nudum* (*Ps.n.*)	Psilotaceae	306 (Pryer *et al.*, 2004)	Herb	0.87											
*Araucaria heterophylla* (*A.h.*)	Araucariaceae	197 (Knapp *et al.*, 2007)	Tree	0.76	0.61										
*Cycas revoluta* (*C.r.*)	Cycadaceae	175 (Magallón and Sanderson, 2005)	Tree	0.69	0.59	0.23									
*Macrozamia riedlei* (*Ma.r.*)	Zamiaceae	175 (Magallón and Sanderson, 2005)	Tree	0.67	0.61	0.23	0.094								
*Ephedra minuta* (*E.m.*)	Ephedraceae	168 (Rydin *et al.*, 2004)	Shrub	1.23	0.85	0.68	0.67	0.70							
*Metasequoia glyptostroboides* (*Me.g.*)	Cupressaceae	146 (Hemsley and Poole, 2004)	Tree	0.87	0.66	0.18	0.27	0.27	0.71						
*Pinus sylvestris* (*Pin.s.*)	Pinaceae	140 (Wang *et al.*, 2000)	Tree	0.80	0.77	0.22	0.30	0.29	0.67	0.25					
*Picea abies* (*Pic.a*)	Pinaceae	130 (Wang *et al.*, 2000)	Tree	0.79	0.69	0.19	0.27	0.26	0.68	0.23	0.080				
*Taxus baccata* (*T.b.*)	Taxaceae	120 (Cheng *et al.*, 2000)	Tree	0.84	0.65	0.15	0.26	0.24	0.73	0.14	0.26	0.22			
*Podocarpus alpinus* (*Po.a.*)	Podocarpaceae	75 (Biffin *et al.*, 2012)	Shrub	0.88	0.71	0.20	0.29	0.29	0.75	0.24	0.32	0.31	0.25		
*Podocarpus nivalis* (*Po.n*)	Podocarpaceae	75 (Biffin *et al.*, 2012)	Shrub	0.87	0.71	0.20	0.28	0.29	0.75	0.24	0.31	0.30	0.24	0.004	
*Cupressus sempervirens* (*Cu.s.*)	Cupressaceae	75 (Stewart, 1993)	Tree	0.88	0.70	0.19	0.25	0.29	0.70	0.085	0.28	0.26	0.16	0.26	0.26

## Results

### Variation in leaf anatomy and morphology in evolutionarily old species

**Fig. 2.**
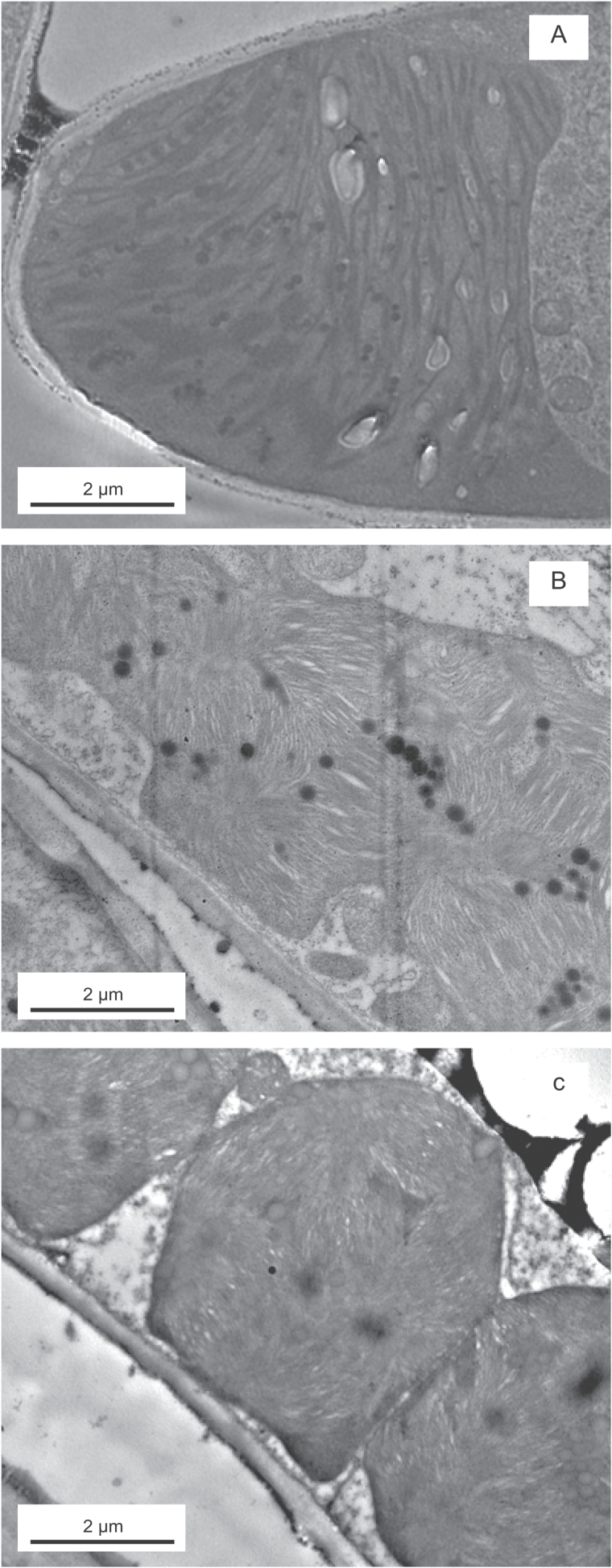
Ultrathin transmission electron microscopy cross-sections showing species with atypical chloroplast shapes and sizes: (A) *Selaginella uncinata*, (B) *Cycas revoluta* and (C) *Macrozamia riedlei*.

**Fig. 3.**
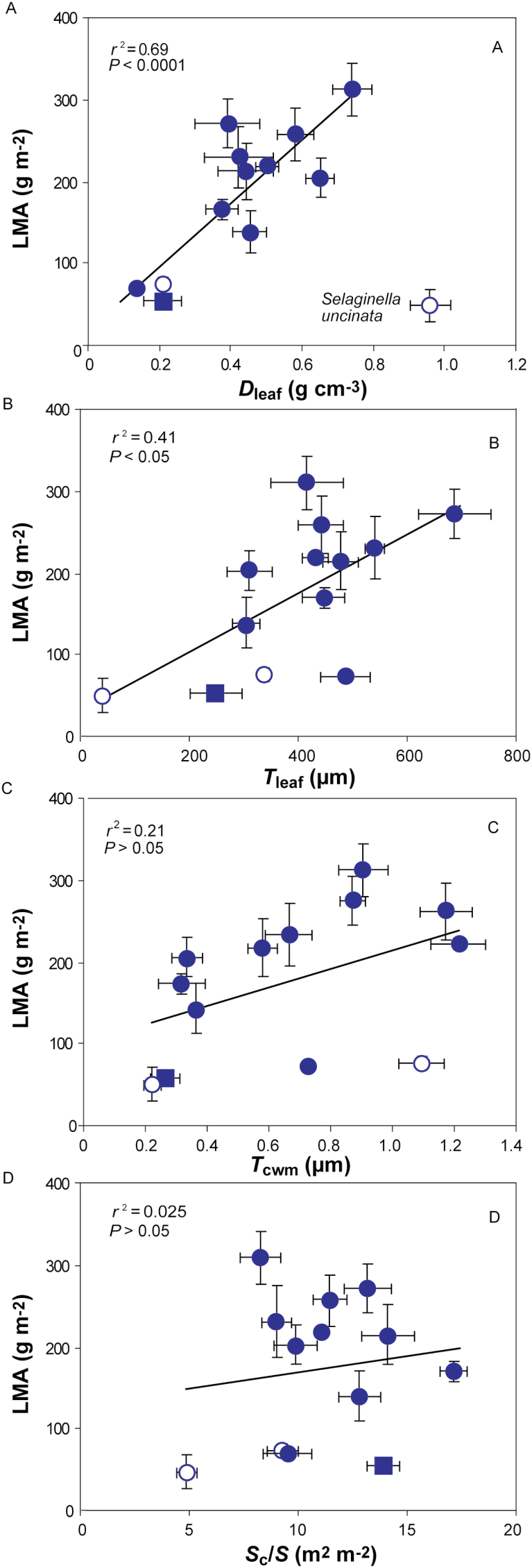
Leaf dry mass per area (LMA) in relation to (A) leaf density (*D*_leaf_), (B) leaf thickness (*T*_leaf_), (C) mesophyll cell wall thickness (*T*_cwm_), and (D) chloroplast surface area exposed to intercellular airspaces (*S*_c_/*S*). Each data point corresponds to one species (*n*=3–5). The non-gymnosperms are marked as open circles, while the only deciduous conifer, *Metasequoia glyptostroboides*, is marked as a filled square. Excluded species are marked with the species’ name. Error bars show average±SE of all presented species. Note that some standard errors are so small that they are not visible under the data points. Data were fitted by linear regression (all are significant at *P*<0.05).

In order to understand the mesophyll diffusional limitations to photosynthesis, a complete structural and ultrastructural analysis was performed of the photosynthetic organs of all the species. A large variability was observed in their macroscopic anatomy (Fig. 1A), but also structural and ultrastructural parameters (Figs 1B–D and 2), e.g. a *P. sylvestris* cross-section exhibited lobed cells, increasing mesophyll surface area exposed to the intercellular airspace (Fig. 1C). LMA varied about 7-fold between species, from 45 ± 12 g m^–2^ in *S. uncinata* to 308 ± 22 g m^–2^ in *C. sempervirens*. Leaf density (*D*_leaf_) and thickness varied 16- and 24-fold, respectively. *T*_cwm_ varied 5.5-fold (from 0.2 ± 0.05 µm in *S. uncinata* to 1.2 ± 0.37 µm in *P. nivalis*), *S*_c_/*S* varied 3.5-fold (from 4.9 ± 0.47 m^2^ m^–2^ in *S. uncinata* to 17.1 ± 0.62 m^2^ m^–2^ in *P. sylvestris*), and *S*_m_/*S* varied 2.9-fold (6.1 ± 0.18 to 17.5 ± 0.72 m^2^ m^–2^). The variation in LMA was attributed to variation in both of its components, but there was a stronger positive relationship with *D*_leaf_ (excluding *S. uncinata* due to its extremely thin leaves) than *T*_leaf_ (Fig. 3A, B). However, there was no correlation between LMA and mesophyll cell wall thickness (Fig. 3C). There was a strong positive relationship between *S*_c_/*S* and *S*_m_/*S* (see Supplementary Fig. S1A), but neither of them was related to LMA (Fig. 3D for *S*_c_/*S*; *r*^2^=0.077, *P=*0.36 for the correlation with *S*_m_/*S*). Similarly, *S*_m_/*S* was not correlated to fraction of intercellular airspaces, *F*_ias_ (*r*^2^=0.007; *P=*0.78). *S*_c_/*S* was unrelated to *T*_mes_ (Supplementary Fig. S1B), but *S*_m_/*S* had a positive correlation (Supplementary Fig. S1C). However, this correlation was not significant when only gymnosperms were included (*r*^2^=0.068; *P=*0.37).

### Interspecific variation in photosynthetic capacity and its relationships with diffusional and structural determinants

Area- and mass-based net assimilation (*A*_mass_) varied about 5- and 8-fold, respectively (from 2.1 ± 0.34 µmol m^–2^ s^–1^ in *P. nudum* to 11.0 ± 0.78 µmol m^–2^ s^–1^ in *P. sylvestris*, and from 21 ± 3.9 nmol m^–2^ s^–1^ in *C. revoluta* to 165 ± 9 nmol m^–2^ s^–1^ in *M. glyptostroboides*). Mesophyll conductance per unit leaf area (*g*_m/area_) varied over 12-fold (from 10 ± 2.1 mmol m^–2^ s^–1^ in *Ephedra minuta* to 124 ± 9 mmol m^–2^ s^–1^ in *P. sylvestris*) and mass-based *g*_m_ (*g*_m/mass_) over 15-fold (from 0.08 ± 0.005 mmol g^–1^ s^–1^ in *P. nivalis* to 1.20 ± 0.06 mmol g^–1^ s^–1^ in *M. glyptostroboides*).

**Fig. 4.**
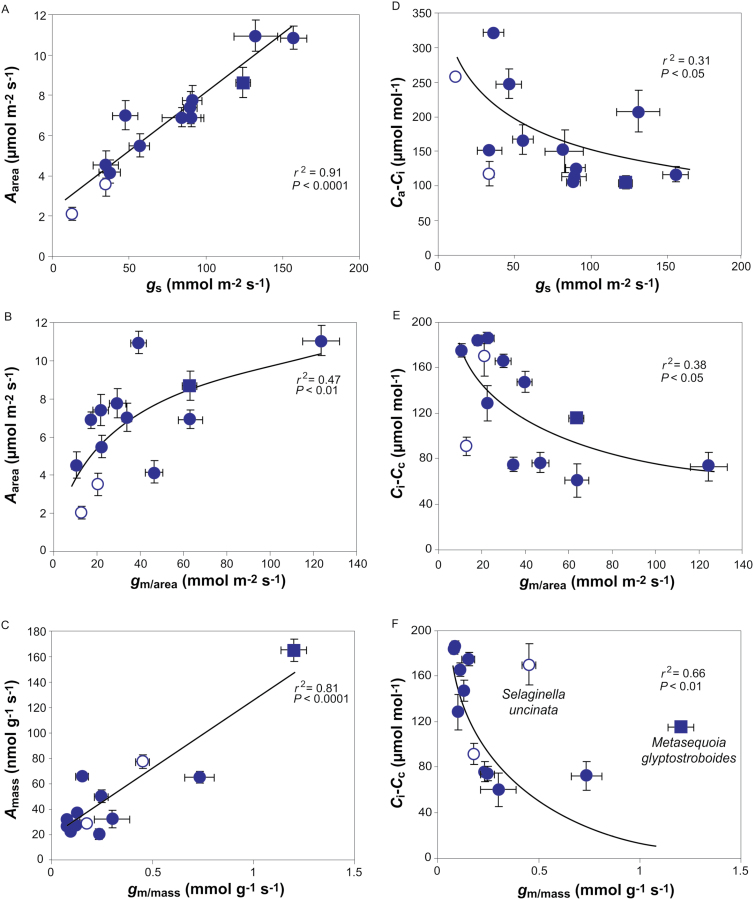
(A, B) Area-based net assimilation (*A*_area_) in relation to (A) stomatal conductance (*g*_s_) and (B) mesophyll conductance per area (*g*_m/area_). (C) Mass-based photosynthesis (*A*_mass_) in relation to mass-based mesophyll conductance (*g*_m/mass_). (D) The draw-down from the atmosphere to the intercellular airspaces (*C*_a_–*C*_i_) in relation to *g*_s_. (E, F) The draw-down from intercellular airspaces to chloroplasts (*C*_i_–*C*_c_) in relation to (E) *g*_m/area_ and (F) *g*_m/mass_. Data in (B) were fitted by non-linear regression of the form *y*=2.73ln(*x*)–2.81; data in (D) of the form *y*=–53ln(*x*)+389; data in (E) of the form *y*=–36ln(*x*)+252; and data in (F) of the form *y*=–30ln(*x*)+80. Data are presented as in Fig. 3.

*A*_area_ depended strongly on *g*_s_ (Fig. 4A). Importantly, *A*_area_ scaled positively with *g*_m/area_ (Fig. 4B), but there was stronger positive correlation between *A*_mass_ and *g*_m/mass_ (Fig. 4C), which was significant even with the exclusion of *M. glyptostroboides* (*r*^2^=0.42; *P=*0.016). A similar strength negative curvilinear relationship was found between *C*_a_–*C*_i_*vs g*_s_ (Fig. 4D) and the CO_2_ drawdown from the intercellular airspace to chloroplasts (*C*_i_–*C*_c_) *vs g*_m/area_ (Fig. 4E). However, a stronger relationship was found between *C*_i_–*C*_c_*vs g*_m/mass_ when *S. uncinata* and *M. glyptostroboides* were excluded (Fig. 4F).

**Fig. 5.**
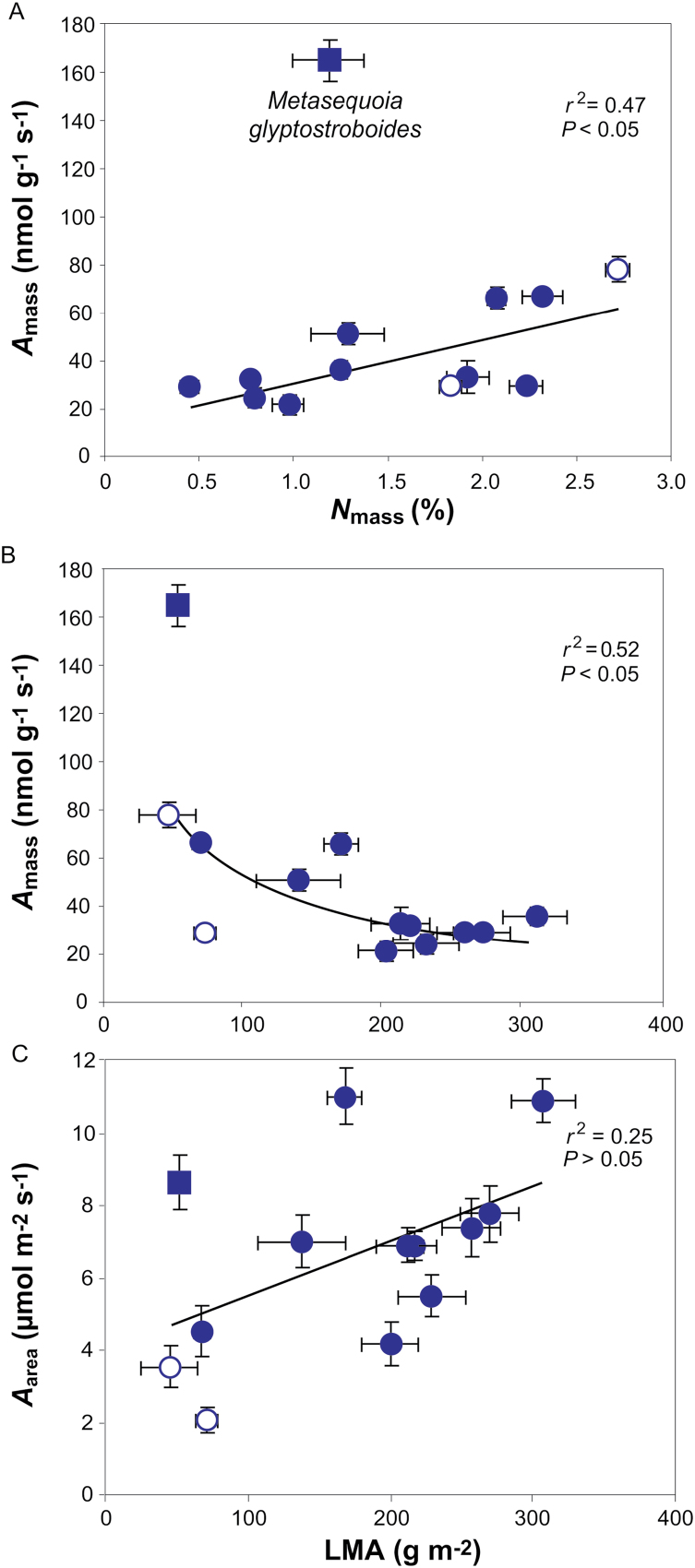
Mass- and area-based net assimilation (respectively *A*_mass_ and *A*_area_) in relation to (A) mass-based nitrogen content (*N*_mas_) and (B, C) LMA. The data in (B) were fitted with a curvilinear regression of the form *y*=896*x*^–0.63^. Data presentation and fitting in (A, C) are as in Fig. 3.

In addition to large variations in LMA and its components, nitrogen content per dry mass (*N*_mass_) varied 6-fold (from 0.45 ± 0.02% to 2.72 ± 0.07%) and area-based nitrogen (*N*_area_) varied 12-fold (0.6 ± 0.01 to 6.1 ± 0.09 g m^–2^). *A*_mass_ scaled positively with *N*_mass_, but the only deciduous conifer in this study, *M. glyptostroboides*, diverged from this relationship by having higher *A*_mass_ (Fig. 5A). There was no relationship between *N*_area_ and *A*_area_ (*r*^2^=0.20; *P=*0.12). Similarly, *A*_mass_ was negatively correlated with LMA, while there was no relationship between *A*_area_ and LMA (Fig. 5B, C).

**Fig. 6.**
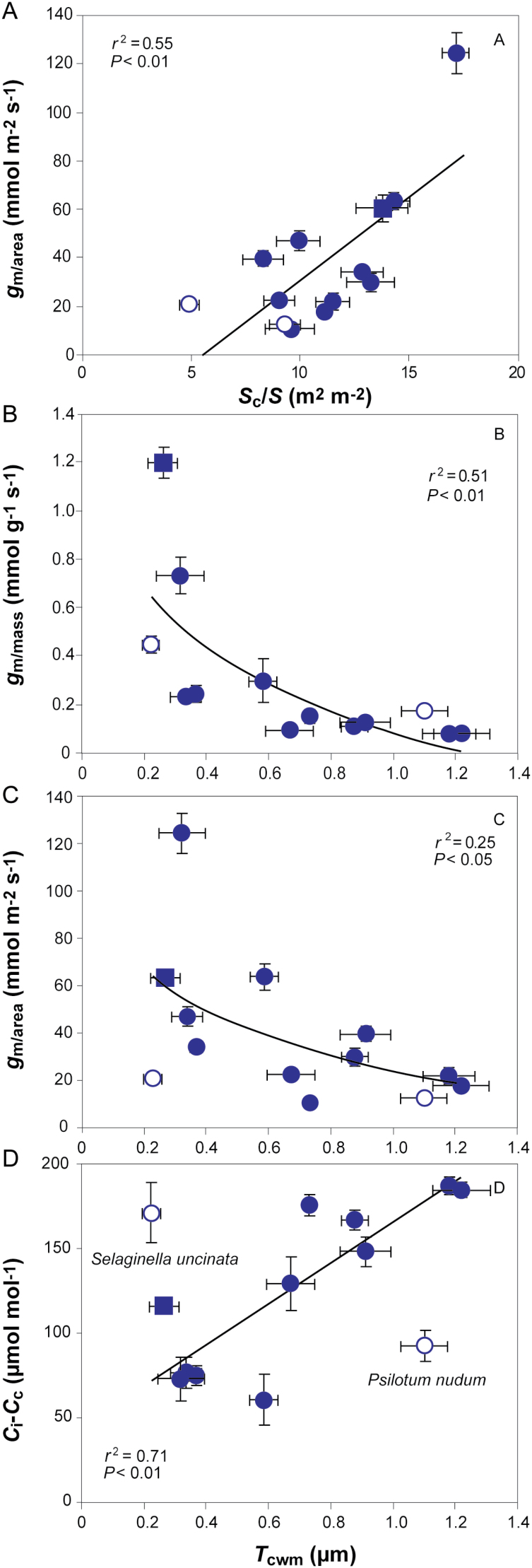
(A) The influence of chloroplast surface area exposed to intercellular airspaces (*S*_c_/*S*) on mesophyll conductance (*g*_m/area_). (B–D) The influence of cell wall thickness (*T*_cwm_) on (B) mesophyll conductance per mass (*g*_m/mass_), (C) mesophyll conductance per area (*g*_m/area_), and (D) the draw-down from intercellular airspaces to chloroplasts (*C*_i_–*C*_c_). The data in (B) were fitted with a non-linear regression of the form *y*=–0.37ln(*x*)+0.09 and data in (C) of the form *y*=–26ln(*x*)+25. Data presentation and fitting are as in Fig. 3.

Analysis of the ultra-anatomical controls on *g*_m_ demonstrated that *g*_m/area_ was positively correlated with *S*_c_/*S*. On the other hand, *g*_m/mass_ and *g*_m/area_ were negatively associated with *T*_cwm_. *C*_i_–*C*_c_ scaled positively with *T*_cwm_ across gymnosperms, but not across the whole sample (Fig. 6). *T*_cwm_ did not correlate with density (*r*^2^=0.030; *P=*0.55).

In addition to gas exchange–fluorescence methodology, *g*_m_ was estimated by two alternative methods: it was modeled from mesophyll anatomical traits by using the anatomical model of Niinemets and Reichstein (2003) and from *A*–*C*_i_ curves (Ethier and Livingston, 2004, Fig. 7). *g*_m_ calculated from anatomical measurements correlated better with the estimates obtained from gas exchange–fluorescence measurements than *g*_m_ obtained from *A*–*C*_i_ curves (average discrepancy between *g*_m_ from gas exchange–fluorescence was 27% with *g*_m_ from anatomy and 33% with *g*_m_ from Ethier and Livingston (2004)). Based on the quantitative limitation analysis, the photosynthetic capacity was strongly limited by both *g*_s_ (range 15–70%) and *g*_m/area_ (range 12–74%), while limited biochemical capacity (range 8–33%) restricted *A*_area_ less than stomata and mesophyll (Fig. 8A). With respect to the structural limitations of *g*_m_, the estimated gas phase limitation inside the leaf was <1% of the total limitations for all the species (data not shown). Among all the components of liquid phase limitations, cytoplasm and membrane limitations played a minor role, whereas the predominant limitation was exerted by cell walls and chloroplast stroma (Fig. 8B and Table 2).

**Fig. 7.**
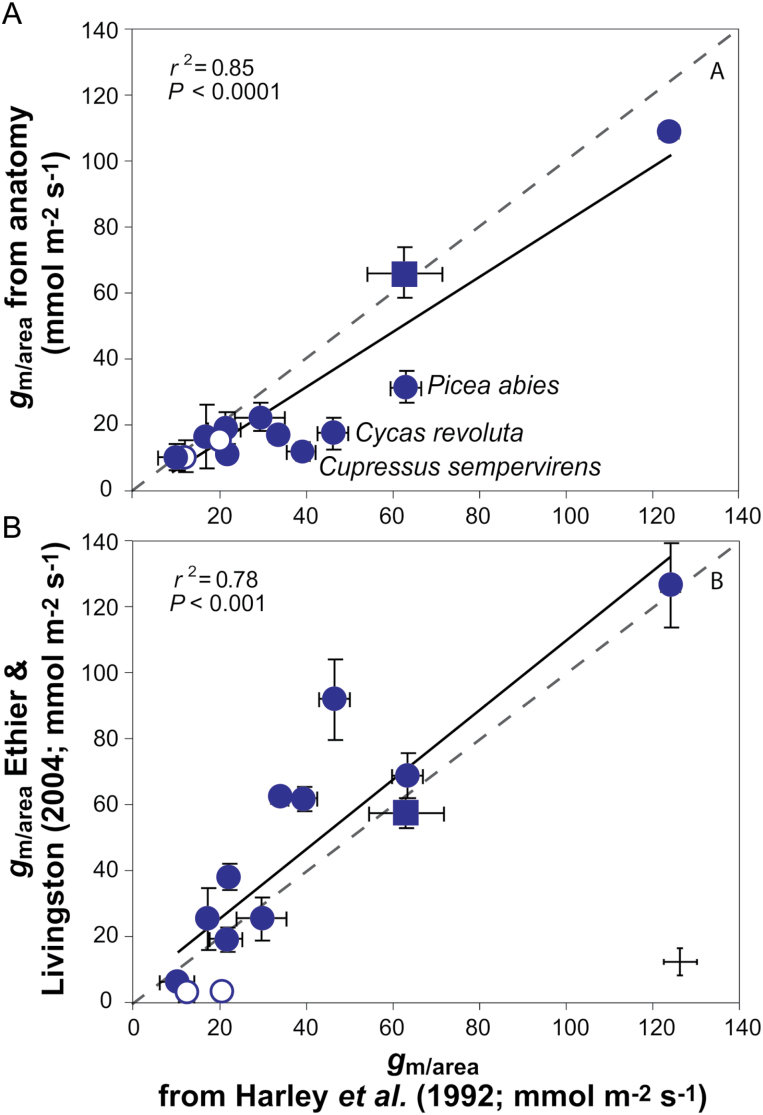
Comparison of *g*_m/area_ calculated based on Harley *et al.* (1992) with (A) *g*_m/area_ calculated from anatomical measurements according to Niinemets and Reichstein (2003) and (B) *g*_m/area_ calculated based on Ethier and Livingston (2004). Dashed lines represent 1:1 correlation. Data presentation and fitting are as in Fig. 3, but all species are included in the analyses. *C. revoluta*, *C. sempervirens* and *P. abies* were underestimated by the anatomical model.

**Fig. 8.**
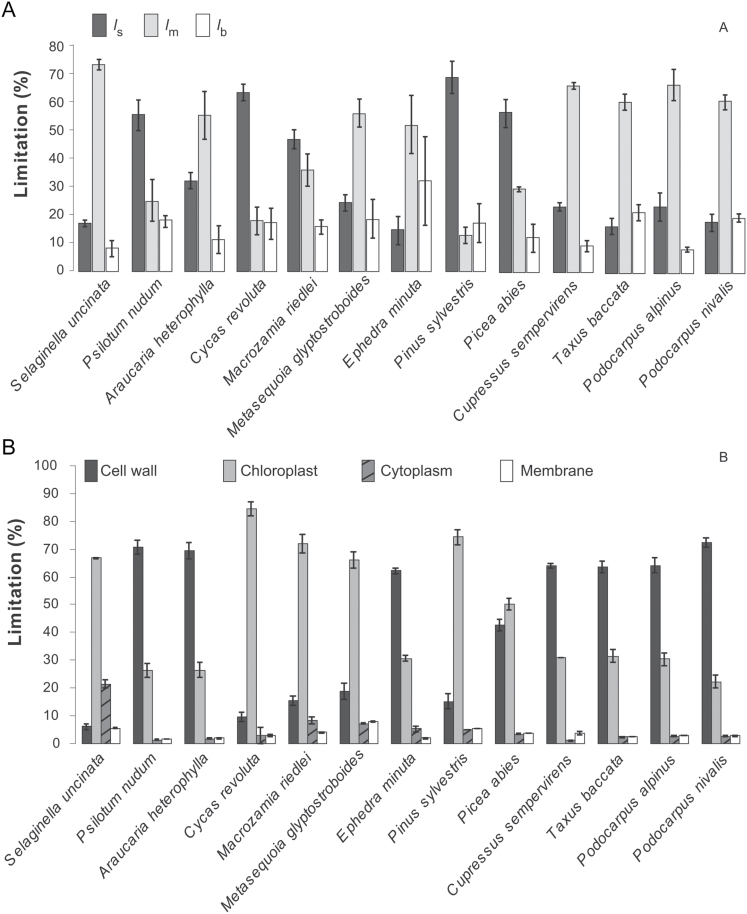
(A) The percentage of assimilation limited by stomatal conductance (*l*_s_), mesophyll conductance (*l*_m_) and biochemistry (*l*_b_). (B) The percentage of mesophyll conductance per area limited by liquid phase components: cell wall, chloroplast, cytoplasm and membrane. Error bars denominate standard errors (*n*=3–5). The species are in the order of evolutionary age.

**Table 2.** *Leaf anatomical parameters* Data are ±SE; *n*=3–5*. F*_ias_, average fraction of intercellular airspace; *T*_cwm_, mesophyll cell wall thickness; *T*_cyt_, cytoplasm thickness; *T*_chl_, chloroplast thickness.

Species	*F*_ias_	*T*_cwm_ (μm)	*T*_cyt_ (μm)	*T*_chl_ (μm)
*Selaginella uncinata*	0.30 ± 0.03	0.22 ± 0.03	0.382 ± 0.033	2.359 ± 0.050
*Psilotum nudum*	0.15 ± 0.01	1.074 ± 0.074	0.177 ± 0.043	4.1 ± 0.3
*Araucaria heterophylla*	0.37 ± 0.09	0.67 ± 0.08	0.09 ± 0.01	2.727 ± 0.092
*Cycas revoluta*	0.20 ± 0.01	0.34 ± 0.05	0.215 ± 0.093	5.36 ± 0.10
*Macrozamia riedlei*	0.20 ± 0.03	0.37 ± 0.02	0.218 ± 0.009	3.918 ± 0.041
*Ephedra minuta*	0.21 ± 0.04	0.731 ± 0.010	0.299 ± 0.065	3.19 ± 0.12
*Metasequoia glyptostroboides*	0.29 ± 0.02	0.26 ± 0.05	0.091 ± 0.020	1.97 ± 0.06
*Pinus sylvestris*	0.21 ± 0.02	0.32 ± 0.08	0.089 ± 0.005	2.25 ± 0.22
*Picea abies*	0.18 ± 0.04	0.58 ± 0.05	0.070 ± 0.024	2.79 ± 0.11
*Taxus baccata*	0.30 ± 0.02	0.876 ± 0.042	0.087 ± 0.003	3.1 ± 0.2
*Podocarpus alpinus*	0.223 ± 0.008	1.18 ± 0.09	0.088 ± 0.009	2.09 ± 0.11
*Podocarpus nivalis*	0.26 ± 0.06	1.22 ± 0.09	0.092 ± 0.002	2.1 ± 0.2
*Cupressus sempervirens*	0.063 ± 0.003	0.91 ± 0.08	0.30 ± 0.09	2.81 ± 0.25

**Fig. 9.**
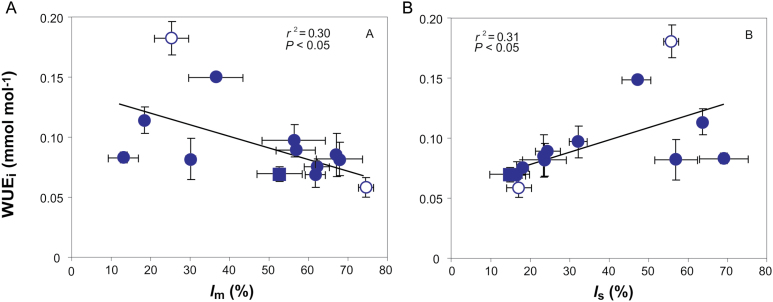
Correlations of intrinsic water use efficiency (WUE_i_) with (A) the share of photosynthesis limited by mesophyll conductance (*l*_m_) and (B) share of photosynthesis limited by stomatal conductance (*l*_s_). Data presentation and fitting are as in Fig. 3.

The intrinsic water use efficiency (WUE_i_) varied *ca* 3-fold across species. It was negatively correlated with the share of photosynthesis limited by *g*_m/area_ (Fig. 9A). WUE_i_ also scaled positively with the photosynthesis limitation by stomata (Fig. 9B). When considered together, the interspecific variation in WUE_i_ was driven by both *l*_s_ and *l*_m_ (see Supplementary Table S2).

### The effect of divergence time on structure and physiology

In the given dataset, LMA and *A*_area_ were negatively correlated with the estimated evolutionary age of the genus (Supplementary Fig. S2 and Supplementary Table S3a; *r*^2^=0.45; *p*=0.012). However, *g*_m/area_ itself was not related to plant evolutionary age (*r*^2^=0.055; *p*=0.44) and *g*_m/area_ influenced *A*_area_ regardless of evolutionary age (Supplementary Table S3b). Nevertheless, the lowest mesophyll conductances were observed for the oldest genera (*Ephedra*: 10.3 mmol m^–2^ s^–1^; *Psilotum*: 12.5 mmol m^–2^ s^–1^; *Selaginella*: 20.5 mmol m^–2^ s^–1^). However, the highest average *g*_m/area_ was recorded for *Pinus* (124 ± 9 mmol m^–2^ s^–1^), although this genus is older than four others (*Picea*, *Podocarpus*, *Taxus*, *Cupressus*) in this study. In addition, *g*_s_ correlated negatively with the age of the genus (*r*^2^=0.41; *P=*0.017), but there was no significant correlation of WUE_i_ with the evolutionary age of the genus (*r*^2^=0.083; *P=*0.34).

## Discussion

### Structural and ultrastructural traits determining LES relationships in evolutionarily old species

LMA in our study depended on leaf density while its relationship with *T*_leaf_ was weaker (Fig. 3A, B) as shown by Tomás *et al.* (2013) across a variety of genera. Contrarily, LMA was more strongly related to *T*_leaf_ than to *D*_leaf_ in Australian sclerophylls (Niinemets *et al.*, 2009*c*). The median value of LMA for evergreen gymnosperms is higher (230 g m^–2^) than what was found here (214 g m^–2^), but the minimum and maximum values here fall into the gymnosperm range (Poorter *et al.*, 2009) placing them at the low end of LES characterized by overall robust foliage and low photosynthetic capacity (Wright *et al.*, 2004).

Prior this study the highest *T*_cwm_ was recorded in ferns and allies: 0.81 µm (Tosens *et al.*, 2016). The species here had even thicker mesophyll cell walls: up to 1.22 µm in *P. nivalis.* Both the average across the sample and across the gymnosperms (0.68 µm) were also very high compared with what has been previously found for non-stressed fully expanded leaves: from approximately 0.1–0.2 µm in annual to 0.4–0.5 µm in sclerophyllous species (Hanba *et al.*, 2001; Terashima *et al.*, 2006; Hassiotou *et al.*, 2009; Tosens *et al.*, 2012*b*, 2016; Tomás *et al.*, 2013). On the other hand, values of *S*_c_/*S* in our study were average in the majority of species as it varies from 1.6 to 32 m^2^ m^–2^ worldwide (Terashima *et al.*, 2006; Peguero-Pina *et al.*, 2012; Tosens *et al.*, 2016) and the lowest values have been recorded in ferns (in the majority of species *S*_c_/*S*<8) (Tosens *et al.*, 2016), but across evergreen gymnosperms *S*_c_/*S* had only been measured in *Abies alba* and *A. pinsapo* (17 and 32 m^2^ m^–2^, respectively) (Peguero-Pina *et al.*, 2012). However, CO_2_ diffusion to Rubisco is greatly enhanced if the cell walls are fully lined with chloroplasts, with *S*_c_/*S*_m_=1, a condition that in practice is rare (Terashima *et al.*, 2011). The ratio was remarkably high in the gymnosperms studied here, being over 0.96 for five species (see Supplementary Fig. S1A).

The LMA components thickness and density alter leaf photosynthetic capacity in opposite directions (Niinemets, 1999). Leaf thickness often reflects a greater number of chloroplasts, higher *S*_c_/*S* and *S*_m_/*S* for CO_2_ diffusion, and greater concentration of photosynthetic machinery, while greater density is associated with lower net assimilation due to increased cell wall resistance (Hanba *et al.*, 1999; Niinemets, 1999; Terashima *et al.*, 2006). Contrarily, Slaton and Smith (2002) studied a wide range of genera, but did not find a correlation between *S*_m_/*S* and *T*_mes_. Similarly, no relationship was found between *S*_c_/*S* or *S*_m_/*S* and *T*_mes_ across Australian sclerophylls (Tosens *et al.*, 2012*b*). However, across the evolutionarily old species studied in this work, *T*_mes_ correlated positively with *S*_m_/*S*, but regardless of the positive trend was not correlated with *S*_c_/*S* (Supplementary Fig. S1B, C). However, the species with high *S*_m_/*S* also had higher *S*_c_/*S* (Supplementary Fig. S1A). This somewhat counterintuitive outcome results from the variability of *S*_c_/*S* due to the spore-bearing plants in the sample as their *S*_c_/*S*_m_ ratio was lower than average. These relationships were not significant, when these two species were excluded from the analyses. *S*_m_/*S* and *S*_c_/*S* depended on mesophyll architecture rather than *T*_mes_ or *F*_ias_ due to variability in density, size and shape of mesophyll cells (e.g. lobes; Fig. 1C) at a given thickness as was previously suggested by Tosens *et al.* (2012*a*). Likewise, this resulted in LMA not correlating with *S*_c_/*S* (Fig. 3D).

Additionally, *T*_cwm_ was not related to LMA (Fig. 3C) or density as is often assumed (e.g. Syvertsen *et al.*, 1995) showing that *T*_cwm_ may not vary with total leaf tissue density or other leaf tissue’s cell wall thicknesses (Tosens *et al.*, 2012*b*). Collectively, these results suggest that widely varying combinations of leaf anatomical traits occur at given values of LMA, and detailed anatomical studies are needed to relate *g*_m_ to mesophyll structure.

### Structural modifications of photosynthesis and mesophyll diffusion conductance

*A*_area_ and *g*_s_ were correlated and comparable to those found in spore-bearing plants and gymnosperms (James *et al.*, 1994; Equiza *et al.*, 2006; Franks, 2006; La Porta *et al.*, 2006; Pardo *et al.*, 2009; Zhang *et al.*, 2015; Tosens *et al.*, 2016). Flexas *et al.* (2012) set an average *g*_m_ slightly above 100 mmol m^–2^ s^–1^ for conifers, and studies have shown a wide variation from as low as 18 up to 110 mmol m^–2^ s^–1^ in evergreen gymnosperms (Warren *et al.*, 2003; De Lucia *et al.*, 2003; Manter and Kerrigan, 2004; Black *et al.*, 2005; Mullin *et al.*, 2009; Peguero-Pina *et al.*, 2012, 2016). Accordingly, *g*_m_ varied from 10 to 124 mmol m^–2^ s^–1^ in this study. The values of *C*_i_–*C*_c_ here fell in the higher part of the range found for angiosperms and were higher than previously found for conifers (Niinemets *et al.*, 2005, 2009*b*; Warren, 2008; Hassiotou *et al.*, 2009; Tosens *et al.*, 2012*b*; Peguero-Pina *et al.*, 2012). Importantly, *g*_m_ influenced both *C*_i_–*C*_c_ and photosynthesis significantly with stronger mass-based relations (Fig. 4).

Nitrogen content was similar to that previously found in conifers and positively related to *A*_mass_, as was also shown by Reich *et al.* (1995). *M. glyptostroboides* diverged in the analysis, which can be explained by its deciduous nature (Wright *et al.*, 2002). A negative relationship between LMA and *A*_mass_ has been shown previously for evergreens while area-based photosynthesis seems not to correlate with LMA, which is contrary to deciduous species, where higher LMA has been shown to correlate with higher *A*_area_ (Reich *et al.*, 1995; Wright *et al.*, 2004). Accordingly, the same was found here (Fig. 5). Altogether, this illustrates strong investment in supportive structure (Niinemets *et al.*, 2009*c*; Hassiotou *et al.*, 2009).

Similarly to recent studies, the most important anatomical correlations with *g*_m_ were *S*_c_/*S* and *T*_cwm_ (Terashima *et al.*, 2011; Tosens *et al.*, 2012*b*, 2016; Peguero-Pina *et al.*, 2012; Tomás *et al.*, 2013) (Fig. 6). The significance of each depends on foliage structure, e.g. in sclerophylls higher *T*_cwm_ overshadows the influence of *S*_c_/*S* on *g*_m_ (Tomás *et al.*, 2013). The *g*_m/mass_–*T*_cwm_ correlation was stronger than the area-based one (Fig. 6B, C) as *g*_m/mass_ indicates the investment in cell walls and *g*_m/area_ in the mesophyll area (Niinemets *et al.*, 2009*b*).

### The importance of structure as a limiting factor of photosynthesis

In order to overcome the various uncertainties related to *g*_m_ estimations, it was additionally modelled from anatomy and estimated from *A*–*C*_i_ curves (Ethier and Livingston, 2004). All three methods show similarly wide variation in *g*_m_ across evolutionarily old species (Fig. 7). However, *g*_m_ from anatomy tended to underestimate, whereas the *A*–*C*_i_ curve method overestimated, *g*_m_ for some species. When both methods were considered, *g*_m_ from the variable *J* method was more strongly correlated with *g*_m_ obtained from anatomy than from *A*–*C*_i_ curves. Likewise, Tomás *et al.* (2013) found this across species exhibiting a wide variation of leaf morphologies. Yet, the model tends to over- or underestimate at the lower or higher end of *g*_m_ (Tosens *et al.*, 2012*a*,*b*; Peguero-Pina *et al.*, 2012; Tomás *et al.*, 2013; Fini *et al.*, 2016). In this sample, *g*_m_ from anatomy mostly underestimated for *C. revoluta*, *C. sempervirens* and *P. abies* (Fig. 7), while the model estimations where in strong agreement with *g*_m_ from gas exchange for species with thick mesophyll cell walls and low *g*_m_. This is different from Tosens *et al.* (2016), where the model overestimated for evolutionarily old *Ophioglossum* and *Lycopodium* species exhibiting very high *T*_cwm_ (0.7–0.81 µm). The discrepancy between measured and modelled *g*_m_ may arise from various uncertainties associated with both estimates. As for combined gas exchange–fluorescence estimation, *g*_m_ is not the only component to affect *C*_c_: the amount of respiratory and photorespiratory CO_2_ should be considered as an independent source of CO_2_ (Tholen *et al.*, 2012). Concerning the predictive power of the anatomical model for species exhibiting a wide variation in *T*_cwm_, heterogeneous chloroplast thickness and morphology (Figs 1 and 2), the strongest uncertainties are related to the unknown variation of mesophyll cell wall porosity and the actual determinants of stroma resistance including the role of carbonic anhydrase (CA). Nobel (1991) proposed an upper value of cell wall porosity of 0.3 and that it varies linearly with *T*_cwm_. Indeed, 0.3 was used to calculate *g*_m_ for *Populus tremula* with low *T*_cwm_ and it gave good results (Tosens *et al.*, 2012*a*). In a multispecies context the relationship between *g*_m_ from anatomy and gas exchange was improved when porosity was modelled varying between 0.04 and 0.095 and being negatively correlated with *T*_cwm_ (Tosens *et al.*, 2012*b*; Peguero-Pina *et al.*, 2012; Tomás *et al.*, 2013). Some species here and in Tosens *et al.* (2016) exhibited very high *T*_cwm_ compared with prior research, and therefore lower porosity values were used for species where *T*_cwm_>0.5 μm. However, as was emphasized by Tosens *et al.* (2012*b*), the linear decline of porosity with *T*_cwm_ is hypothetical and the discrepancy between modelled and measured *g*_m_ could be resolved by some other trait.

Quantitative limitation analysis further confirmed that the mesophyll gas phase limitation to CO_2_ diffusion is negligible as it accounted for less than 1% of the total limitation. This is even less than previously observed in a wide range of sclerophyllous angiosperm genera or in spore-bearing plants (Tosens *et al.*, 2012*a*,*b*; Tomás *et al.*, 2013). Using limitation analysis to further separate the contribution of the components of the liquid phase revealed that the limitation of *T*_cwm_ and *T*_chl_ had the greatest span, ranging from 6 to 72% and from 22 to 84%, respectively (Fig. 8B). This is in agreement with the negative correlation between *g*_m/mass_ and *T*_cwm_ (Fig. 6B). CO_2_ faces the longest diffusion distance in the liquid phase after entering the chloroplasts (Evans *et al.*, 2009). In this set of species, *T*_chl_ varied between 1.97 and 5.36 μm (Fig. 2), which is thicker than previously reported explaining the high limitation by *T*_chl_. It is debated whether the CO_2_ diffusion efficiency in the chloroplast stroma is mainly controlled by CA activity in the chloroplast (Flexas *et al.* 2012) and if it varies across species (at normal cytosolic pH), being higher in evergreen sclerophylls in order to compensate for the low CO_2_ diffusion efficiency through thick mesophyll cell walls (Gillon and Yakir, 2000), or whether CA is always sufficient and CO_2_ interconversion is not a limiting factor of *g*_m_. Equally, this analysis adds to the evidence that CO_2_ diffusion is importantly controlled by the physical diffusion distance within the chloroplasts (Tosens *et al.*, 2012*a*,*b*; Peguero-Pina *et al.*, 2012; Tomás *et al.*, 2013).

Overall, the good fit between *g*_m_ calculated from anatomy and gas exchange confirms that *g*_m_ is explained to a large extent by inherent variations in mesophyll anatomy, while mesophyll cell walls and chloroplasts are the most important structural determinants of liquid phase conductance in species exhibiting high *T*_cwm_, *T*_chl_ and heterogeneous chloroplast morphology. However, the limitation amplitude was large implicating versatility in its importance among different species (Fig. 8A). The smallest limitation on *A*_area_ on average was biochemistry, which is similar to previous interspecies studies by Tomás *et al.* (2013) and Tosens *et al.* (2016). As the mesophyll and stomatal conductances were low, especially compared with maximal values measured so far (Wright *et al.*, 2004; Flexas *et al.*, 2012), it is expected that biochemistry plays a smaller role in limitations, because the photosynthetic enzymes are not saturated with CO_2_ due to constraints on the previous parts of the pathway (Terashima *et al.*, 2011; Niinemets *et al.*, 2011). The limitations by stomata and mesophyll were similarly significant in the control of WUE_i_ showing again that leaf mesophyll structure plays an important role in realized WUE_i_ (Fig. 9).

### The effect of divergence time on structure and physiology

The rate of photosynthesis depended on the age of the genera. This, however, was mostly due to the inclusion of spore-bearing plants reflecting the evolutionary increase in photosynthetic capacity from early plants to angiosperms (Brodribb *et al.*, 2009; Carriquí *et al.*, 2015; McAdam and Brodribb, 2015). However, when broad taxonomic groups are considered, species’ evolutionary adaptation to light and water conditions can actually drive photosynthetic capacity more strongly than their evolutionary age (Tosens *et al.*, 2016). In this regard, it is important that *A*_area_ depended on *g*_m/area_ positively regardless of evolutionary age, showing that this correlation remained significant including the effect of divergence time in the model (see Supplementary Table S3a, b).

Analogously to *A*_area_, it has been suggested that there is an increase in *g*_m/area_ through the evolution and diversification of embryophytes (Carriquí *et al.*, 2015). While indeed gymnosperms studied here had a very low average *g*_m/area_, much lower than angiosperms with comparably tough foliage structure (e.g. Flexas *et al.*, 2012), *g*_m/area_ did not significantly depend on evolutionary age. As with within-group variability in ferns (Tosens *et al.*, 2016) and angiosperms (Flexas *et al.*, 2007*b*), this likely reflects individual species adaptation to specific habitat conditions.

Notwithstanding the lack of within-group evolutionary signal, low average *g*_m/area_ and *A*_area_ and the corresponding thick mesophyll cell walls for species with widely contrasting ecological strategies do support the preservation of old traits through evolution, suggesting apparent constraints on evolution. Different CO_2_/O_2_ selection pressures at the time of divergence for ferns and angiosperms have been hypothesized to be the reason for lower average *A*_area_ values for the former (Carriquí *et al.*, 2015) as ferns evolved in several-fold higher CO_2_ and slightly lower O_2_ concentrations than angiosperms (Brodribb *et al.*, 2009; Carriquí *et al.*, 2015). In fact, in the high-CO_2_ atmosphere where several of the thick-cell-walled species evolved (about 65–200 My ago), diffusional limitations exercised a lower control on the rate of photosynthesis. However, while this ancient trait has been preserved through evolution, *g*_m_ has started to exercise an increasingly stronger control on foliage assimilation rates. Yet, this might be inevitable given the inefficient dynamic stomatal control under water-limited conditions potentially leading to excess plant water loss until hydraulic constraints force stomatal closure (Brodribb *et al.*, 2005; Carnicer *et al.*, 2013).

## Conclusions

This study demonstrates important relationships between mesophyll structure and physiology in evolutionarily old plants. A large variability was found in ultrastructural traits determining LES relationships. Although LMA depended on leaf density and thickness, they were not correlated with *T*_cwm_ or *S*_c_/*S* illustrating that LMA cannot be used as a universal explanation for photosynthetic structural constraints.

Mesophyll anatomy exerted major control on net assimilation rates. The three methods used in previous studies for interspecies comparisons were used to confirm *g*_m_ values from gas exchange–fluorescence measurements and their anatomical nature. Although high variation was uncovered between species in *g*_m_, it was on average very low. This resulted mainly from the exceptionally thick cell walls, large chloroplasts, and low *S*_c_/*S*. The role of stroma deserves further attention.

These characteristics in evolutionarily older taxa support the preservation of ancient traits in evolution, although the role of functional adaptation and evolutionary constraint in leaf anatomy continues to be debated.

## Supplementary Material

Supplementary Data

## Abbreviations:

*A*_area_: net assimilation rate per area
*A*_mass_: net assimilation rate per dry mass
C_a_: atmospheric CO_2_ concentration
CA: carbonic anhydrase
*C*_a_–*C*_i_: CO_2_ drawdown from atmosphere to intercellular airspace
C_i_: intercellular CO_2_ concentration
*C*_i_–*C*_c_: CO_2_ drawdown from intercellular airspace to chloroplasts
*D*_leaf_: leaf density
*F*_ias_: fraction of intercellular airspace
LMA: leaf dry mass per area
*l*_b_, *l*_m_ and *l*_s_: photosynthetic limitation by biochemistry, mesophyll and stomatal conductance, respectively
*N*_area_: nitrogen content per area
*N*_mass_: nitrogen content per mass
*g*_m_: mesophyll diffusion conductance
*g*_m/area_ and *g*_m/mass_: mesophyll conductance per area and per mass
*g*_s_: stomatal conductance
*S*_c_/*S* and *S*_m_/*S*: chloroplast and mesophyll area exposed to intercellular airspace
*T*_chl_: chloroplast thickness
*T*_cwm_: mesophyll cell wall thickness
*T*_leaf_: leaf thickness
*T*_mes_: mesophyll thickness
WUE_i_: intrinsic water use efficiency.
