# Supplementary material for: Extremely thick cell walls and low mesophyll conductance: welcome to the world of ancient living!
Source: J Exp Bot. 2017 Apr 13;68(7):1639–53. doi: 10.1093/jxb/erx045 (PMC5441924; doi:10.1093/jxb/erx045)
Supplement: Supplementary Data [file erx045_Supplementary_Data.zip › supplementary_figures_S1_S2_Tables_S1_S3.pdf]

Supporting Information

Article title: **Extremely thick cell walls and low mesophyll conductance: welcome to the world of ancient living!**

Authors: Linda-Liisa Veromann-Jürgenson[1], Tiina Tosens[1], Lauri Laanisto[1], Ülo Niinemets[1,2]

The following Supporting Information is available for this article:

Fig. S1 a) Correlation between chloroplast surface area exposed to intercellular airspaces (S_c/S) and mesophyll surface area exposed to intercellular airspaces (S_m/S). b) Correlation between chloroplast surface area exposed to intercellular airspaces (S_c/S) and mesophyll thickness (T_{mes}). c) Correlation between mesophyll surface area exposed to intercellular airspaces (S_m/S) and mesophyll thickness (T_{mes}).

Data presentation and fitting are as in Fig. 3.

Fig. S2 Correlation between leaf mass per area (LMA) and evolutionary age (in millions of years, My). Data presentation and fitting are as in Fig. 3.

Table S1 The habitat and the origin of plants or seeds of the studied species.

Species	Climate	Range of dispersal	Origin of plants or seeds[1]
Selaginella uncinata	Subtropics to tropics	China	GC
Psilotum nudum	Mediterranean to tropics	Global	GC
Araucaria heterophylla	Subtropics to tropics	Norfolk Island	GC
Cycas revoluta	Warm temperate to subtropics	Japan	GC
Macrozamia riedlei	Mediterranean	Southwestern Australia	Kings Park, Crawley, Perth, Western Australia (31.97º S, 115.83º E)
Ephedra minuta	Temperate to subtropics	China	GC
Metasequoia glyptostroboides	Warm temperate	China	GC
Pinus sylvestris	Boreal to temperate	Eurasia	Saare parish, Estonia (58.70º N, 26.84º E)
Picea abies	Boreal to temperate	Eurasia	Saare parish, Estonia (58.70º N, 26.84º E)
Taxus baccata	Temperate	Europe, northern Africa, Iran, southwestern Asia	GC
Cupressus sempervirens	Mediterranean	Mediterranean Europe, Western Asia	Bellver forest, Mallorca, Spain (39.56º N, 2.62º E)
Podocarpus alpinus	Temperate	Southern Australia	GC
Podocarpus nivalis	Temperate	New Zealand	GC

The species are order of decreasing evolutionary age.

[1] GC - gardening center (Jardin, Tartu; Bauhof, Tartu; Nurga Nursery, Harjumaa), the origin of seeds and seedlings in the gardening centers are the Netherlands and Germany.

Table S2. Dependence of the intrinsic water use efficiency (WUE_i) on limitations by mesophyll conductance ($g_{m/area}$) and limitation by stomatal conductance (g_s).

	SS	df	Direction	F	*P*	*Partial η^2
Intercept	0.052	1		54.0	<0.0001	0.84
Limitation by g_s (l_s)	0.014	1	Negative	15.1	0.003	0.60
Limitation by $g_{m/area}$ (l_m)	0.011	1	Negative	11.4	0.007	0.53
Error	0.010	10				

*Partial η^2 is the variance explained by a given variable of the variance remaining after excluding variance explained by the other predictors. The analysis demonstrates that the interspecific variation in WUE_i was driven by both l_s and l_m, but the effect was somewhat stronger for l_s.

Table S3. Dependence of net assimilation rate on evolutionary age (all effects are significant at $P < 0.05$).

a) Effect of evolutionary age of the genera on A_{area}.

Effect	df	SS	r^2	*P*
Evolutionary age	1	47.7	0.46	0.011
Error	11	5.12		

b) Effect of evolutionary age and $g_{m/area}$ on A_{area}.

Effect	df	SS	*AIC*	*P*
Evolutionary age	1	28.4	52.8	0.014
$g_{m/area}$	1	24.7	52.8	0.019
Error	10	31.7		

Fig. S1.

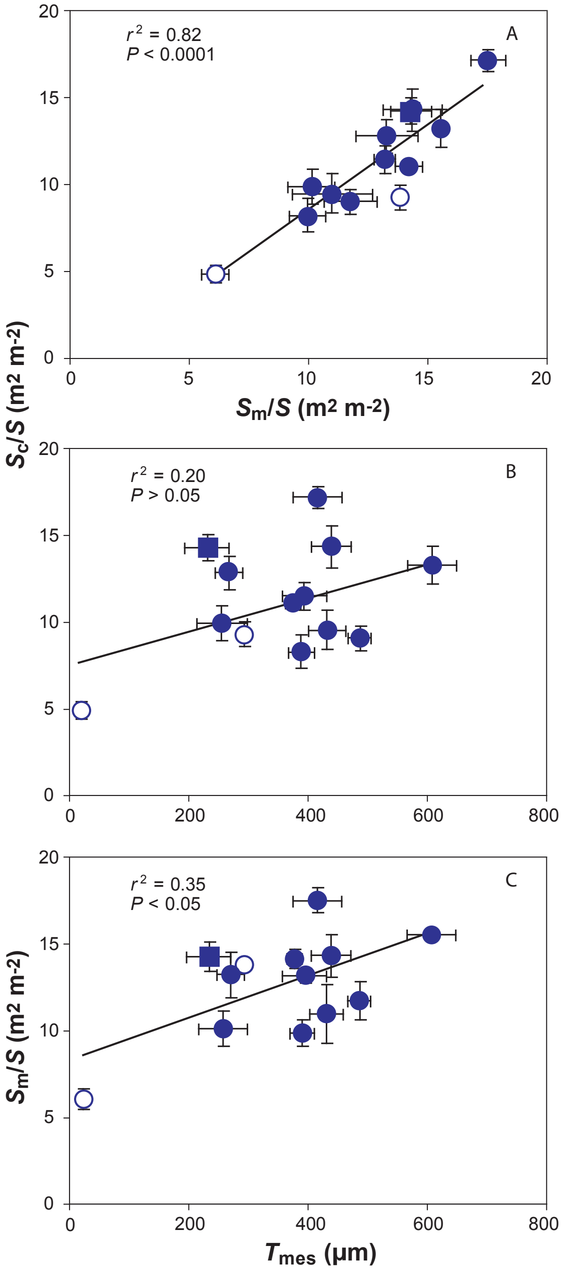
r^2 = 0.82
P < 0.0001
A
S_c/S (m2 m-2)
S_m/S (m2 m-2)
r^2 = 0.20
P > 0.05
B
r^2 = 0.35
P < 0.05
C
S_m/S (m2 m-2)
T_{mes} (µm)

Fig. S2.

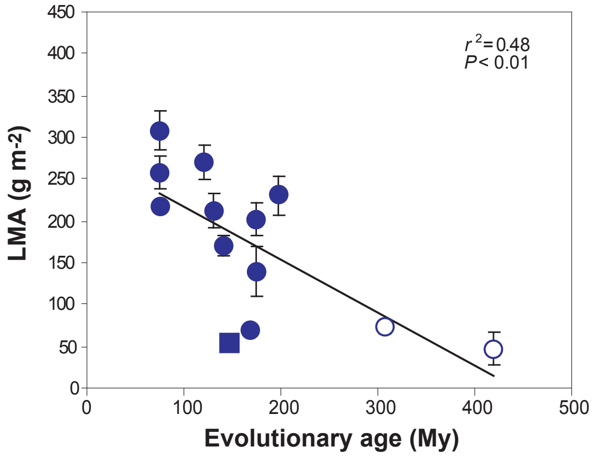

450
400
350
300
250
200
150
100
50
0
0
100
200
300
400
500
LMA (g m-2)
Evolutionary age (My)
r 2= 0.48
P< 0.01
